# Neoadjuvant endocrine therapy in breast cancer: current role and future perspectives

**DOI:** 10.3332/ecancer.2016.609

**Published:** 2016-01-07

**Authors:** Romualdo Barroso-Sousa, Danilo DA Fonseca Reis Silva, Joao Victor Machado Alessi, Max Senna Mano

**Affiliations:** Instituto do Câncer do Estado de São Paulo (ICESP), Faculdade de Medicina, Universidade de São Paulo, Av Dr Arnaldo, 251, 5o andar, 01246-000 São Paulo, SP, Brazil

**Keywords:** breast neoplasm, luminal breast cancer, locally advanced breast cancer, neoadjuvant therapy, endocrine therapy, aromatase inhibitors

## Abstract

Luminal breast cancer, as defined by oestrogen and/or progesterone expression by immunohistochemistry, accounts for up to 75% of all breast cancers. In this population, endocrine therapy is likely to account for most of the gains obtained with the administration of adjuvant systemic treatment. The role of adjuvant chemotherapy in these patients remains debatable since it is known that only a small fraction of patients will derive meaningful benefit from this treatment whilst the majority will be exposed to significant and unnecessary chemotherapy-related toxicities, in particular the elderly and frail. Therefore, neoadjuvant endocrine therapy (NET) becomes an attractive option for selected patients with hormonal-receptor positive locally advanced breast cancer. In this review, we discuss the current role of NET and future perspectives in the field.

## Introduction

Despite continuous scientific progress, patients diagnosed with locally advanced breast cancer (LABC)–here defined as stage IIB, IIIA, and IIIB cancer according to TNM classification [1], remain at high risk of recurrence and death from breast cancer (BC). Data from the surveillance epidemiology and end results (SEER) report five year survival rates of 70%, 52%, and 48% for patients presenting with stage IIB, IIIA, and IIIB disease respectively [2].

For these patients, current standard treatment is neoadjuvant chemotherapy (NCT) followed by surgery, adjuvant radiotherapy, and endocrine therapy to those with tumours expressing hormonal receptors. Phase III randomised trials showed that NCT is safe and at least equivalent to adjuvant chemotherapy in terms of disease-free and overall survival (OS) [3]. Furthermore, NCT induces tumour downstaging and increases rates of breast-conserving surgery (BCS) [4, 5]. However, factors other than tumour stage alone should be considered in the treatment plan of a patient with LABC, including 1) patient-related (age, global functional status, comorbidities, medications, social aspects, and patient preferences); 2) disease-related, in particular tumour biology–currently best defined as the molecular subtype of BC.

Luminal tumours, as defined by oestrogen receptor (ER) and/or progesterone receptor (PgR) expression by immunohistochemistry (IHC), account for 75% of all BC, summing up to 85% in women over 70 years old [6]. In this population, adjuvant endocrine therapy (AET) is likely to account for most of the gains obtained with the administration of adjuvant systemic treatment and the need for additional adjuvant chemotherapy in these patients remains debatable. In the Oxford meta-analysis, absolute OS benefits with polychemotherapy versus nil in postmenopausal women with HR+ disease are no higher than 3–4%, in contrast with more than 10% for the comparison of tamoxifen versus nil [7, 8]. Further support for this view is provided by recent studies of genetic signatures–in which the majority of women with luminal cancers have low-risk scores and do not derive meaningful benefit from the addition of chemotherapy to AET. These conclusions apply to both women with node negative [9, 10] and node positive disease [11]. Conversely, the majority of these patients will be exposed to significant and unnecessary chemotherapy-related toxicities, in particular the elderly and frail [12]. It is recognised that short and long term toxicity can become an issue especially in patients with an excellent prognosis–in particular the risk of myelodysplastic syndrome, non-lymphocytic leukaemia, and heart disease [13, 14].

The key role of AET in patients with luminal BC provides the main rationale for the investigation of NET in this population. Different groups have investigated the role of this treatment modality, in addition to biomarkers of response in this setting [15, 16]. In this review, we discuss the current role of NET and future perspectives in the field.

## Primary endocrine therapy: to whom?

Endocrine therapy emerged in the early 1980s as a treatment option for elderly women who were unfit to be treated with chemotherapy or ineligible for surgery [17]. These studies were designed to evaluate the role of tamoxifen as a primary treatment option as an alternative to surgery rather than as a neoadjuvant treatment. Subsequently, trials were designed to compare primary endocrine therapy (PET) with tamoxifen versus surgery (with or without AET) and a meta-analysis of seven clinical trials failed to show difference in OS (hazard ratio HR: 0.98; P = 0.9), but found that patients treated with surgery did experience gains in terms of progression-free survival (PFS) (HR: 0.55; P = 0.0006) [18]. However, an important limitation of these trials was the lack of ER status for the majority of the patients, as only one study selected patients according these biomarkers [19]. Based on these results, we recommend that PET should only be offered to women with HR+ tumours who are unfit for surgery or refuse this procedure, or to elderly women with short life expectancy as established by a qualified specialist and based on a validated geriatric assessment tool.

## Neoadjuvant aromatase inhibitors × tamoxifen: which is the best choice?

The third-generation aromatase inhibitors (AI) anastrozole, letrozole, and exemestane are currently considered standard treatment for women with early or advanced BC, based on clinical trials that demonstrated their superiority over tamoxifen [20–23]. In the neoadjuvant setting, four phase III randomised clinical trials addressed this same question–three in postmenopausal and one in premenopausal women (Table 1) [24–27].

P024 was a multinational, double-blind trial that randomly assigned 337 women with LABC (clinical stage II or III) who were ineligible for BCS to treatment with four months of letrozole (2.5 mg daily) or tamoxifen (20 mg daily), followed by surgery [28]. Letrozole was found to be superior to tamoxifen in terms of clinical response rate as assessed by palpation (55% versus 36%, P = 0.001), ultrasound (35% versus 25%, P = 0.042), mammography (34% versus 16%, P = 0.001), and also in terms of breast conservation rate (45% versus 35%, P = 0.022) [29]. Immediate preoperative anastrozole, tamoxifen, or combined with tamoxifen (IMPACT) was a phase III, double-blind, multicentre trial that randomised 330 postmenopausal women with HR+ operable or potentially operable LABC to anastrozole, tamoxifen, or a combination of tamoxifen and anastrozole for 12 weeks before breast surgery [25]. Clinical response rate based on caliper for anastrozole, tamoxifen, and the combination were 37%, 36%, and 39% respectively, whilst the ultrasound response rates were 24%, 20%, and 28%. There were no significant differences between the treatment arms either by caliper or ultrasound measurement. In the 124 patients considered to require mastectomy at baseline, an improved rate of BCS was reported in patients treated with anastrozole (44% versus 31%), although this difference was not statistically significant (P = 0.23). On the other hand, 46% and 22% of the patients receiving anastrazole and tamoxifen respectively, were deemed by the surgeon to have achieved tumour regression sufficient to allow BCS, which was statistically significant (P = 0.03). However, not all patients accepted this recommendation.

The preoperative anastrozole compared with tamoxifen (PROACT) trial was a randomised, multicentre, double-blind study comparing three months of anastrozole to tamoxifen as preoperative treatment of postmenopausal women with large, operable (T2/3, N0-2, M0), or potentially operable (T4b, N0-2, M0) ER+ and/or PgR+ invasive BC [26]. In this study, a total of 451 women were enrolled, and at the investigator’s discretion concurrent NCT was permitted, with such treatment eventually being given to 29% of the patients treated with anastrozole and 32% of those treated with tamoxifen. There was no significant difference in overall response rate between patients who received anastrozole or tamoxifen either by caliper (50.0% versus 46.2%, P = 0.37 respectively) or ultrasound measurement (39.5% versus 35.0% P = 0.029 respectively). In patients who did not receive chemotherapy and who were not considered candidates for BCS at study entry, the objective response rates for anastrozole and tamoxifen were respectively 48.6% and 35.8% as measured by calipers (P = 0.04) and 36.6% and 24.2% as measured by ultrasonography (P = 0.03). In this subgroup of patients, treatment with anastrazole improved surgical options (43.0% versus 30.8%, P = 0.04). In summary, anastrozole proved to be at least as effective as tamoxifen in this study and was probably more effective in certain clinically relevant selected through clinically relevant subgroups, which strengthens the conclusion that AI are superior to tamoxifen in the neoadjuvant setting.

Finally, a Russian study compared exemestane with tamoxifen in 151 women with HR+ T2-4, N0-2 BC, both given for three months. Clinical response rates were higher with exemestane (76.3% versus 40%; P = 0.05), though ultrasound and mammographic response were not different. Higher rates of BCS were reported in the exemestane arm (36.8% versus 20.0%; P = 0.05) [30].

A meta-analysis of these studies, including a total of 1160 patients indicated–as expected–superior outcomes in terms of clinical objective response rate (RR 1.29; 95% CI, 1.14–1.47; *P* < 0.001), ultrasound response rate (RR, 1.29; 95% CI, 1.10–1.51; P = 0.002), and BCS rate (RR, 1.36; 95% CI, 1.16–1.59; *P* < 0.001) with AI as compared to tamoxifen. Furthermore, there was no difference in clinically relevant toxicities between the two treatments [31].

In premenopausal women, data from the Austrian Breast and Colorectal Cancer Study Group Trial 12 (ABCSG-12) established that three-year adjuvant treatment with anastrozole plus the gonadotropin-releasing hormone (GnRH) agonist goserelin is associated with similar outcomes to adjuvant tamoxifen plus goserelin [32]. Conversely, recent data from two international collaborative group trials–the suppression of ovarian function trial (SOFT) and the tamoxifen and exemestane trial (TEXT)–depicted a significant absolute improvement of 3.8% in disease-free survival (DFS) with five years of suppression of ovarian function and exemestane, as compared with five years of ovarian suppression and tamoxifen [33]. Hence, in the premenopausal (adjuvant) setting, the combination of AI and ovarian suppression was shown to be either superior or at least as good as tamoxifen and ovarian suppression. The tamoxifen or Arimidex combined with goserelin acetate to compare efficacy and safety. A (STAGE) trial which was a phase III, randomised, double-blind multicentre study was undertaken that addressed this same question in the preoperative setting. This trial allocated 197 premenopausal women with HR+, HER-2-negative BC with operable and measurable lesions (T [2–5 cm], N0, M0) to anastrozole 1 mg daily or tamoxifen 20 mg daily for 24 weeks, both given in combination with goserelin [27]. The results confirmed that anastrozole was superior to tamoxifen in terms of caliper response (70.4% versus 50.5%, P = 0.004), ultrasonography response (58.2% versus 42.4%, P = 0.027), and magnetic resonance imaging (MRI) or computed tomography (CT) response (64.3% versus 37.4%, P = 0.032). Also, more patients in the anastrozole group had BCS compared with the tamoxifen group (86% versus 68%). These data suggest that anastrozole plus goserelin is an effective neoadjuvant treatment option in this patient population. In summary, even though the role of NET in premenopausal women remains largely investigational, the results of the STAGE trial are consistent with the findings of major adjuvant studies and it could be expected that the superior activity of neoadjuvant AI would translate into improved cancer outcomes with continued treatment in the adjuvant setting.

## Which aromatase inhibitor?

ACOSOG-Z1031 was the only study prospectively designed to compare exemestane, letrozole, and anastrozole head-to-head in the neoadjuvant setting [34]. This was a phase II trial that recruited 377 postmenopausal women with clinical stage II or III (T2-T4c, N0-3, M0), HR+ (Allred score ≥ 6) disease to receive one of the three AI for four months before surgery. No statistically significant differences in clinical response (62.9% exemestane × 74.8% letrozole × 69.1% anastrozole) or surgical outcomes were reported in this study (Table 1). These results, interpreted in conjunction with the previously described trials comparing neoadjuvant AI to tamoxifen and additional data from studies in the adjuvant and metastatic settings, suggest that the effectiveness of the three commercially available AIs are largely equivalent.

## What is the optimum duration of neoadjuvant endocrine treatment?

Based on previous experience with NCT, a three to four-month duration of NET has been proposed in the majority of the clinical trials such as P024 [24], IMPACT [25], and PROACT [26]. However, evidence from other studies suggests that three to four months duration of NET could be insufficient to achieve maximum reduction in tumour volume.

Dixon *et al* retrospectively investigated the potential benefits of prolonged treatment with neoadjuvant letrozole in 182 patients with operable or locally advanced HR+ BC [35]. Of the 182 patients, 63 were continued on letrozole beyond three months because of different reasons: a total of 26 responded but had not responded sufficiently to allow BCS, 15 patients responded but still had inoperable disease, 13 were unfit and considered unsuitable for surgery, and 9 refused surgery. The response rate in the 182 patients increased from 69.8% at three months to 83.5% after prolonged treatment. Importantly, the BCS rate increased from 60% to 72%. Few women whose tumours initially responded to letrozole had disease progression after three months of therapy. Reductions in tumour volume were observed at all time periods (52% during the first 3 months, 50% from month 3–6, 37% from month 6–12, and 33% from month 12–24), indicating sustained letrozole activity. However, because of the retrospective nature of the data, it is important to highlight the possibility of a selection bias in this population.

A Spanish phase II trial aimed to establish the optimal duration of treatment with neoadjuvant letrozole in an open-label study that included 70 postmenopausal women with a mean age of 79 years [36]. This study revealed a median time to objective response of 3.9 months and a median time to maximal response (i.e. time until the lesion had reached stabilisation at evaluation) of 4.2 months. More than a third of the responders (37.1%) achieved maximal reduction in tumour volume after six months. No additional responders were recorded after eight months of treatment though. Another recently published phase II trial with neoadjuvant exemestane also demonstrated an increase in overall clinical response as measured by clinical palpation from 58.7% at three months to 68.3% at the last palpation measurement (>three months) [37].

More robust data was provided by a recent longitudinal phase IV, multicentre, prospective, open-label study that treated 146 patients with early BC, initially unsuitable for BCS, with letrozole therapy for a maximum of 12 months or until they became eligible for BCS, progressed, failed to meet criteria for BCS, and withdrew for scheduled mastectomy [38]. The median time to achieve a tumour response sufficient to allow BCS with neoadjuvant letrozole was 7.5 months (95% CI 6.3–8.5 months), which also supports the conclusion that extended neoadjuvant AI therapy (to beyond three to four months) might be required to achieve maximal reduction in tumour volume or at least sufficient reduction to allow for BCS. Importantly, only nine (6.5%) patients in this trial had disease progression during extended NET. In the previously discussed STAGE trial, there was an increase of 13.3% and 9.1% in tumour responses from week 16 to week 24 in the anastrozole and tamoxifen arms respectively, providing additional evidence that treatment duration of at least 24 weeks is closer to optimal in this setting.

It is currently unknown if treatment extension to beyond 12 months could further improve response, but there is a theoretical risk that genomic events such deleterious resistance-inducing mutations would start to arise during the treatment, triggering the event of tumour progression and even disease spread beyond the breast [27]. In the studies discussed above usually we see when evaluating treatment durations of up to 6–12 months, the rates of disease progression during extended therapy were low (0.8–12.0%) [34, 39, 40]. We suggest that until further data becomes available, the optimal duration of NET should be individualised based on careful and serial evaluation of clinical response and patient’s clinical status. In our practice, we tend to treat patients for at least six months (or at least no less than four months). Beyond six months, we tend to continue NET until maximal response or up to the point where BCS becomes possible–always a decision to be taken in conjunction with the surgical team.

## Neoadjuvant endocrine therapy versus neoadjuvant chemotherapy: what is the evidence?

There is limited data comparing NCT to NET, the best evidences coming from two randomised phases II trials. In one study, 239 postmenopausal women with stage IIA–IIIB HR+ BC were randomised to receive preoperative AI (anastrozole or exemestane for three months) or chemotherapy (four, three week cycles of doxorubicin 60 mg/m^2^ plus, paclitaxel 200 mg/m^2^) [41]. There was no statistically significant difference between AI and chemotherapy in terms of clinical response rate, time to response, or pathologic complete response (pCR). Similarly, GEICAM/2006-03 randomised 97 patients with IHC-defined luminal disease (ER+/PR+/HER-2-/cytokeratin 8/18+) to receive neoadjuvant exemestane for 24 weeks or chemotherapy (four, three-week cycles of epirubicin 90 mg/m^2^ plus cyclophosphamide 600 mg/m^2^ followed by four, three–week cycles of docetaxel 100 mg/m^2^) [42]. As this trial permitted the inclusion of premenopausal women, this subgroup of patients treated in the exemestane arm also received goserelin for castration. Although no statistically significant difference was found between the two arms in terms of clinical response rate (48% × 66% for exemestane and chemotherapy respectively; P = 0.075), there was a trend for a worse outcome in the exemestane arm for premenopausal patients and those with high tumour Ki67 expression (luminal B).

The NEOCENT trial (neoadjuvant chemotherapy versus endocrine therapy), which was designed to compare NCT (FEC100: epirubicin, 5-fluorouracil, cyclophosphamide) to NET (letrozole) in postmenopausal women with strongly HR+ primary BC, was unfortunately prematurely closed because of slow accrual, and it is therefore unlikely to contribute in clarifying this critically important question [43].

## Are there any validated biomarkers to predict short (response) and/or long-term (recurrence/death) outcome?

In NCT studies, pCR is considered a validated endpoint of long term outcomes, especially in more biologically aggressive subtypes such as triple negative and HER-2 positive BC [44]. However, in patients with luminal BC treated with NCT, the end point appears to be less useful because of the low frequency of pCR in this setting. Interestingly, the prognostic/predictive value of pCR after NCT also appears to be lower in luminal than in HER-2 positive or triple negative BC, suggesting the implication of factors other than simply the low frequency of pCR [44]. In addition, even with a careful selection of patient candidacy for NET, a small fraction of them will develop disease progression on treatment [24, 25]. Therefore, the identification of powerful biomarkers of short and long-term response to NET is a matter of great interest.

One interesting candidate parameter is the Ki-67 antigen which is expressed during all cell-cycle phases except for G_0_, and is considered a surrogate of cell proliferation [45]. Although there is continued (and probably justified because of the lack of methodology standardisation) resistance to adopt Ki-67 in the routine practice, some international consensuses such as ESMO and Saint Gallendo support its role in distinguishing between the two intrinsic subtypes of HR+ BC namely luminal A and B [46, 47]. Assuming that the action of endocrine therapy in BC is mainly because of inducing cell-cycle arrest, an on-treatment Ki-67 can be considered a potential surrogate of response to endocrine therapy. Interestingly, on-treatment levels of Ki-67 in relatively small NET were apparently able to anticipate outcomes from large adjuvant trials that showed the superiority of AI over tamoxifen in postmenopausal women [23, 25, 48, 49] (Table 2). Data from STAGE trial also support the role of on-treatment Ki-67 measurement for predicting the outcomes from large adjuvant studies in premenopausal women [27, 33] (Table 2). In addition in ACOSOG-Z1031, in line with the main results of the study, there was no difference in Ki67 decrease between letrozole, anastrozole, and exemestane, and again on-treatment Ki67 analysis was reportedly able to predict the primary endpoint of the trial i.e. it suggested that there were no statistically significant differences in tumour response between the three AIs [34]. Finally, on-treatment Ki67 results from phase II ACOSOG-Z1031 were able to anticipate outcomes from the large adjuvant trial MCIC CTG MA.27 [50] (Table 2).

It is however important to highlight that the best time point to assess Ki-67 response is not well defined. In the IMPACT trial–in which Ki-67 was measured as early as two weeks–the superiority of anastrozole over tamoxifen was confirmed based on a greater reduction in Ki-67 levels either after two and 12 weeks (P = 0.013 and P = 0.0006 respectively), despite the fact that the trial was negative for its primary endpoint (clinical response) [51]. Furthermore, in a multivariate analysis, Ki-67 expression at two weeks was significantly associated with recurrence-free survival (HR = 1.95; P = 0.004) [52]. One potential limitation of this very early assessment of Ki-67 is the development of delayed or acquired resistance. Paradoxical increases in Ki67 in the surgical specimen compared with baseline (greater than 5%) were seen in 12.3% and 5.8%, of luminal A and luminal B patients respectively included in ACOSOGZ1031 trial [34]. In addition in IMPACT trial, the authors showed that 15% of patients who had early (week two biopsy) decreases in Ki67 on NET had increases in Ki67 at the time of surgery (as assessed on surgical specimens), raising doubts about the optimal time-points for assessment of this and other potential biomarkers [53]. Currently, the advantage of measuring two-week Ki67 instead of pretreatment Ki67 is being prospectively investigated in the large (*n* = 4.000) peri-operative endocrine therapy for individualising care (POETIC) window-of-opportunity trial [53].

An interesting prognostic tool was developed based on data from 228 tumour samples of patients from the P024 trial, namely the PEPI (the preoperative endocrine prognostic index) score, which is based on the analysis of several features in the tumour specimen after NET, including pathological tumour size, node status, ER status, and Ki-67 levels (Table 3). Patients with PEPI score 0 were considered to have had good response to NET and had an excellent long-term outcome in this study. The PEPI score was subsequently validated in the IMPACT trial population [54]. More recently, additional biomarker analyses of ACOSOG Z1031 showed that 27.1% and 10.7% of IHC-defined luminal A and luminal B cancers respectively achieved a PEPI score of 0. Furthermore luminal A cancers showed greater Ki-67 full response (as defined by a drop to ≤1%) at week 2–4 biopsies as compared to luminal B (32.9% versus 16.7%, P = 0.001) [34]. In summary, in these two small cohorts of patients, a low risk PEPI score (in particular a ‘0 score’) was associated with an excellent outcome and these patients are unlikely to benefit from additional chemotherapy. Taken together, these data imply that patients with less proliferative tumours will eventually be the greater responders to endocrine therapy as expected.

In order to further evaluate these concepts, the ongoing ALTERNATE trial (also known as Alliance A011106 trial) is designed to recruit 2280 postmenopausal women with clinical stage II or III HR+, HER-2-negative BC to receive NET with anastrozole, fulvestrant, or the combination of these two drugs for 24 weeks. Tumour biopsies for Ki-67 assessment will be performed at the end of week four and 12. If Ki-67 is found to be >10%, patients are recommended to switch to 12 weeks of weekly paclitaxel for the determination of the pCR in this population. Patients with Ki-67 <10% will continue their assigned regimen to complete 24 weeks of NET followed by surgery and PEPI score calculation. Hopefully, this trial will help to definitively validate the PEPI-0 status as marker of excellent prognosis with endocrine therapy alone, as additional adjuvant chemotherapy is not being recommended for these patients (ClinicalTrials.gov identifier: NCT01953588).

Also interesting is the German ADAPT umbrella study that aims to evaluate the role of Ki-67 as an early predictive surrogate marker for therapy response under a short induction treatment [55]. One of its sub-studies, the ADAPT HR+/HER-2- (ClinicalTrials.gov identifier: NCT01779206), is designed to recruit 4000 HR+, HER-2 negative BC women between 18 and 75 years of age with any tumour size (T1-T4, except inflammatory BC) and nodal status (N0-N3). All patients will receive a three week induction preoperative endocrine therapy based on currently AGO guidelines (i.e., premenopausal: tamoxifen (20 mg, daily); postmenopausal: AI (letrozole (2.5 mg, daily), anastrozole (1 mg, daily), or exemestane (25 mg, daily)) at investigator’s choice). IHC evaluation of Ki-67 will be determined by central pathology and the measurements will be performed from the diagnostic core biopsy tumour sample and the repeat core biopsy after induction therapy. Optimal therapy response is defined as a drop of Ki-67 to or below 10%. Besides the Ki-67 value after the induction therapy, treatment decision will be based on pretreatment risk assessment (recurrence score (RS) by the OncotypeDx^®^ and nodal status). In ADAPT trial, any N0-1 patient will also obtain an Oncotype DX^®^. Patients with RS 0–11 are classified as low-risk and are further treated just by AET according to AGO guidelines. In the intermediate-risk group (N0-1 and RS 12–25), patients with Ki-67 ≤10% are considered to be sufficiently treated by AET alone. Non responders (post-therapeutic Ki-67 >10%) and patients initially identified as high-risk for recurrence (N2-3 or N0-1 and RS ≥ 26) will be randomised to a chemotherapy protocol optimising dose-dense taxane-based chemotherapy.

Of note, genomic alterations have increasingly become the focus of investigation in this setting. In a recent study, Ueno and colleagues evaluated the role of OncotypeDx 21-gene assay in predicting clinical response to NET. In this trial, core biopsy-derived samples from patients treated with exemestane for 24 weeks were analysed for standard IHC-based prognostic factors and the recurrence score (RS) [56]. The clinical response rate in patients with a low RS was significantly higher than in patients with a high RS (59.4% versus 20.0%, respectively; P = 0.015) and similar to patients with an intermediate RS (59.4% versus 58.8%, respectively). Rates of BCS were 90.6% (29/32), 76.5% (13/17), and 46.7% (7/15) in the low, intermediate, and high RS groups respectively. There was a significant difference in average reduction in tumour size between low and high RS group (31.8% versus 12.5% [*p* = 0.045]). More recently, Turnbull and coworkers analysed pre- and on-treatment (after two weeks and three months) biopsies from 89 postmenopausal women with ER+ BC treated with letrozole as NET for transcription profiling [57]. A four-gene signature was able to predict clinical response with an accuracy of 96%. This signature is based on the pretreatment level of two genes (one gene [IL6ST] is associated with immune signalling, and the other [NGFRAP1] with apoptosis) and the on-treatment (two-week) level of two proliferation genes (ASPM, MCM4). This signature also significantly predicted recurrence-free survival (P = 0.029) and breast cancer specific survival (P = 0.009). The four-gene signature was found to be 91% accurate in an independent validation data set. The authors also demonstrated that the test can be performed by quantitative polymerase chain reaction or IHC, which greatly facilitates future clinical application of this biomarker. Although these data are considered promising and may even indicate that the future of response prediction in endocrine therapy belongs to modern genomic signatures, caution is warranted in interpreting these results because of the small sample size and non-randomised and retrospective nature of these studies.

## Future perspectives

Genomic analyses have disclosed alterations in key cancer pathway components in luminal BC, including receptor tyrosine kinases, PI3K-AKT-mTOR, RAS-RAF-MAPK, and p53-RB, which leads to cell cycle progression and resistance to cell death [58]. Clinical trials have tested different compounds targeting these altered pathways. These efforts started to pay off in recent years with two novel compounds incorporated to the armamentarium of HR+ BC treatment.

The PI3K-AKT-mTOR pathway has been shown to be the most frequently altered signalling pathway in BC, and robust data have shown significant crosstalk between the ER and PI3K-AKT-mTOR pathways making the latter one of the most important alterations leading to endocrine resistance. Therefore, targeting this pathway was a logical step. Actually, mTOR inhibition became one of the recent success stories in the treatment of luminal BCs. In 2012, everolimus an mTOR inhibitor, was approved by Food and Drug Administration (FDA) and European Medicines Evaluation Agency (EMEA) for the treatment of postmenopausal women with advanced HR+/HER2- BC in combination with exemestane after failure of either letrozole or anastrozole. The approval of everolimus was based on the results of BOLERO-2 trial that showed that the combination was associated with a robust and statistically significant improvement in PFS compared with exemestane alone (median PFS 7.8 versus 3.2 months; HR = 0.45; *P* < 0.001), although no significant improvement in OS improvement was reported (median OS 31.0 versus 26.6 months; not significant) [59, 60]. The neoadjuvant setting helped to pave this path after a phase II randomised clinical trial demonstrated that the combination of everolimus and letrozole improved clinical and proliferative (Ki-67 decrease) response warranting further investigation of everolimus combined with endocrine therapy in luminal BC [61]. Currently, at least three studies are investigating the role of different inhibitors of PI3K-AKT-mTOR pathway, using the neoadjuvant model as a clinical development platform (Table 4).

As previously shown by genomic analyses studies, the cyclin-CDKs-RB pathway is also altered in luminal BC and appears to be associated with endocrine resistance [62]. Cyclin-dependent kinases (CDKs) are critical regulatory enzymes that regulate all cell cycle transitions, making them interesting targets in BC [63]. Recently, palbociclib, a selective inhibitor of CDK4-CDK6 kinases, given in combination with letrozole received conditional approval by the FDA for the first line treatment of postmenopausal women with HR+/HER-2- advanced BC. The approval of palbociclib was granted on the basis of the impressive results of the PALOMA-1, which showed an improvement in PFS favouring the combination (median PFS 20 versus ten months; HR = 0.32; *P* < 0.001) [64]. Currently, there are at least two studies investigating the role of palbociclib in combination with AI as neoadjuvant therapy in women with HR+ BC (Table 4). Large adjuvant studies addressing the role of PI3K-AKT-mTOR and CDK4-6 inhibitors are also planned or already ongoing.

It is also necessary to highlight that these new agents can be associated with increased toxicity as compared to endocrine therapy alone, which in a way will undermine the elegance of endocrine therapy, usually considered a very well tolerated treatment. The incorporation of these agents in the early BC scenario will eventually be a matter of careful evaluation of the risk/benefit ratio. Careful evaluation of the cost-effectiveness of these agents is also likely to become an issue in many countries [59, 64, 65].

Of note, how PgR expression affects the effectiveness of ET is a matter of debate. As the PgRgene expression is regulated by ER, it is assumed that a lack of expression of PgR indicates a non-functional ER pathway [66]. However, whether this information defines a subgroup with limited benefit from endocrine therapy is controversial [7, 67]. A recent study added information on the biological role of PgR in the ER pathway in breast tumours [68]. The authors showed that PgR associates with ER to direct ER chromatin binding events within breast cancer cells, resulting in gene expression associated with good prognosis. Furthermore, progesterone was able to inhibit oestrogen-mediated growth of ER cell line xenografts and primary ER breast tumour explants and increase anti-proliferative effects when combined with an ER antagonist. This data supports the investigation of combinations of traditional endocrine agents with PgR agonists.

Finally, it is important to mention that neoadjuvant design is a very interesting approach to drug development in BC. The traditional model based on large phase III adjuvant studies has limitations, especially given the long time for the final results to become available and growing costs of these trials. As previously mentioned, using on-treatment Ki67 values as an endpoint on a relatively small NET trials, we were able to accurately predict outcomes of large adjuvant trials, both in post and premenopausal women (Table 2). Another example of how neoadjuvant design can accelerate the development of new drugs or sometimes simply refine the use of old compounds is the NEWEST trial [69]. This trial compared fulvestrant 500 versus 250 mg in the neoadjuvant setting and anticipated the superiority of fulvestrant 500 mg as demonstrated in the phase III randomised CONFIRM trial in metastatic setting [70] (Table 2). Therefore, it is our view that future phase III trials in HR+ BC should take into account data generated by well-conducted NET studies.

## Conclusions

Considering the existing data on NET discussed in this review and also given many uncertainties, one questions what is finally the right place of NET in current clinical practice? Probably the most suitable patients are postmenopausal women, in particular (but not limited to) older women, ideally with low-grade HR-rich (Allred ≥6 for both ER and PR) luminal A cancers. In addition, patient preferences, geriatric assessments, and comorbidities should all be taken into consideration to ensure that NET is the most suitable treatment in a particular situation. Additionally, because of their well-established role in predicting benefit from adjuvant endocrine therapy, genomic signatures can be occasionally used as a tool to gain deeper insights about tumour biology–a low risk result can boost confidence that NET is the right choice to a given patient.

There is currently insufficient data to recommend the routine use of early biopsies to guide treatment, but a large trial addressing this question is underway. Importantly, one should keep in mind that the decision to prescribe NET to a given patient is not necessarily an irreversible one; in case of poor clinical response, the treatment can be switched to immediate surgery or chemotherapy. As a note of caution, despite NET being indeed a unique opportunity to measure endocrine responsiveness ‘*in vivo*’, current data provide insufficient evidence to dismiss the need for adjuvant chemotherapy in patients with good response to NET, especially in fit women with advanced stage cancers. Results of ongoing clinical trials such as ALTERNATE–which will hopefully validate the use of PEPI score as surrogate marker of excellent outcome with ET alone–are eagerly awaited by the scientific community. In the meantime, the decision to offer additional chemotherapy to women exposed to NET should be individualised.

Whist there is clarity regarding the superiority of AIs over tamoxifen in this setting, many uncertainties remain about the optimal duration of NET. Until additional research sheds light into this issue, we recommend at least six months of treatment (unless a response plateau is reached at an earlier point) and usually up to a maximum 12 months, with even longer treatment durations allowed in selected cases.

## Figures and Tables

**Table 1.** Randomised clinical trials comparing different endocrine agents in the neoadjuvant setting.

Trials profile				Study population	Outcomes		
**Trial (reference)**	**Treatment arm (n)**	**Phase**	**Duration**	**Characteristics of populations**	**Primary endpoint**	**OR**	**Downstaging to BCS**
**P024** [13]	A: Letrozole (162) B: Tamoxifen (175)	IIb–III	4 months	ER+ and/or PgR+ ≥10% Postmenopausal Staging: T2-4a-c, N0-2, M0^α^	OR by clinical palpation	A: 55% B: 36% P < 0.001^#^	A: 45% B: 35% P = 0.022
**IMPACT** [14]	A: Anastrazole (113) B: Tamoxifen (108) C: Tamoxifen + anastrazole (109)	III	12 weeks	ER staining ≥1% Postmenopausal Operable or potentially operable BC^β^	OR by caliper	A: 37% B: 36% C: 39%	A: 44% B: 31% C: 24% P = 0.23
**PROACT** [15]	A: Anastrozole (228) B: Tamoxifen (223)	III	3 months	ER+ and/or PgR+ Postmenopausal Operable or potentially BC^γ^	OR by ultrasound	A: 39.5% B: 35.4%	A: 43.0%^*^ B: 30.8%^*^ P = 0.04
**Russian trial** [20]	A: Exemestane (76) B: Tamoxifen (75)	NA	3 months	ER+ and/or PgR+ Postmenopausal T2-4, N0-2, M0	OR by clinical palpation	A: 76.3% B: 40.0% P = 0.05	A: 36.8% B: 20.0% P = 0.05
**STAGE** [16]	A: Anastrozole + goserelin (98) B: Tamoxifen + goserelin (98)	III	24 weeks	ER staining ≥10% + HER-2 negative Premenopausal Operable and measurable lesions T2, N0, M0	OR by caliper	A: 70.4% B: 50.5% P = 0.004	A: 86% B: 68%
**ACOSOG** **Z1031** [24]	A: Exemestane (124) B: Letrozole (128) C: Anastrozole (125)	II	16–18 weeks	ER with Allred score of 6–8 Postmenopausal T2-T4c, N0-3, M0	OR by clinical palpation	A: 62.9% B: 74.8% C: 69.1%	A: 48.1%^∞^ B: 42.1% C: 64%

BC – breast cancer; BCS – breast conservative surgery; ER – oestrogen receptor; HR – hormonal receptor; OR – objective response rate; PgR – progesterone receptor; NA – not available.

* In improved feasible surgery in hormone therapy only group (n = 314).

# by clinical palpation.

α None were BCS candidates at baseline; 14% deemed inoperable.

β Pretreatment surgical assessment available for 220 patients–96 eligible for BCS.

γ 386 of the patients either required a mastectomy or were deemed inoperable at baseline.

∞ Among candidates for mastectomy only at presentation.

**Table 2.** Clinical utility of on-treatment Ki67 level measured in NET trials at different time-points.

Study^#^ (n with available Ki-67 data)	Time of Ki67 assessment	Clinical utility^#^
P024 [19] (185)	4 months	Predicted the results of BIG 1–98 trial (n = 8.010) [39] (letrozole > tamoxifen)
IMPACT [14] (259)	2 weeks	Predicted the results of ATAC trial (n = 9.366) [38] (anastrozole > tamoxifen)
ACOSOGZ1031 [24] (266)	16–18 weeks	Predicted the results of MA27 trial (n = 7.576) [40] (anastrozole = exemestane)
STAGE [16] (188)	24 weeks	Predicted the results of SOFT/TEXT trials (n = 4.690) [23] (anastrozole + goserelin > tamoxifen + goserelin)
NEWEST [55] (211)	4 weeks	Predicted the results of CONFIRM trial (n = 736) [56] (fulvestrant 500 mg > fulvestrant 250 mg)

# References. NET– neoadjuvant endocrine therapy; n = number of patients.

**Table 3.** The preoperative endocrine prognostic index (PEPI) score and validation studies.

Pathology biomarkers status after NET	Risk points for RFS
**Tumour size**	
T1/T2	0
T3/T4	3
**Node status**	
Negative	0
Positive	3
**Ki67 level**	
0–2.7%	0
>2.7–7.3%	1
>7.3–19.7%	1
>19.7–53.1%	2
>53.1%	3
**ER status, Allred score**	
0–2	3
3–8	0
**PEPI risk groups and recurrence rate**	
**Discovery set, P024 trial^+^ (n = 158)**	
0	10%
1–3	23%
4^+^	48%
**Validation set, IMPACT trial^#^ (n = 203)**	
0	3%
1–3	5%
4^+^	17%

+ Median follow-up of 62 months,

# Median follow-up of 37 months.

ER – oestrogen receptor, NET – neoadjuvant endocrine therapy, PEPI – preoperative endocrine prognostic index. RFS – recurrence-free survival; n = number of patients.

**Table 4.** Ongoing clinical trials of NET plus PI3K/AKT/mTOR inhibitors or (CDK) 4/6 inhibitors.

Register number (N)	Design	Arms and regimen	Duration	Primary outcome
**PI3K inhibitor**				
NCT02273973 N = 330	Phase II randomised, double-blind, placebo-controlled trial Randomisation 1:1	Arm A: Taselisib (6 mg: 5 days on, 2 off) plus letrozole Arm B: Placebo + letrozole	16 w	pCR
NCT01923168 N = 360	Phase II randomised, double-blind, placebo-controlled trial Randomisation 1:1:1	Arm A: BYL719 (300 mg q.d.) + letrozole Arm B: Buparlisib (100 mg q.d.) + letrozole Arm C: Placebo + letrozole	24 w	pCR
**AKT inhibitor**				
NCT01776008 N = 87	Phase II, open label, single-arm trial	MK-2206 (q.d. on days 2, 9, 16 and 23) + anastrozole; goserelin acetate on day one of each cycle (if premenopausal)	Maximum four cycles of 28 days each	pCR based on Ki67 values
**CDK4/6 inhibitor**				
NCT01723774 N = 29	Phase II, open label, single-arm trial	PD0332991 (on days 1–21 of each 28 day cycle) + anastrozole + goserelin acetate on day 1 of each cycle (premenopausal patients only	Maximum four cycles of 28 days each	Complete cell cycle arrest based on Ki67 values
NCT0229801 N = 306	Phase II randomised, open label Randomisation 3:2:2:2	Arm A: Letrozole (14 w) Arm B: Letrozole (2 w) then letrozole + palbociclib^*^ (12 w) Arm C: Palbociclib^*^ (2 w) then letrozole + palbociclib^*^ (12 w) Arm D: Letrozole + palbociclib^*^ (14 w)	14 w	Change in Ki67 values cCR
NCT02400567 N = 132	Phase II randomised, open label Randomisation 1:1	Arm A: Chemotherapy (three cycles of FEC100 then three cycles of docetaxel 100 mg/m^2^) Arm B: Letrozole + palbociclib^*^ (12 w)	18 w	RCB 0–I index rate

* Palbociclib dose – 25 mg capsule orally daily for a three weeks on and one week off cycle, BC – breast cancer, cCR – clinical complete response, CDK – cyclin-dependent kinase, ECOG-PS – Eastern Cooperative Oncology Group performance status, FE –5-fluorouracile 500 mg/m^2^, epirubicin 100 mg/m^2^, cyclophosphamide 500 mg/m^2^), HR – hormonal receptor, ORR – objective response rate, pCR – pathological complete response; RCB – residual cancer burden, w – weeks.

## References

[1] Sobin LH, Gospodarowicz MK, Wittekind C (2010). International Union against Cancer. TNM classification of malignant tumours.

[2] Newman LA (2009). Epidemiology of locally advanced breast cancer. Semin Radiat Oncol.

[3] Mauri D, Pavlidis N, Ioannidis JP (2005). Neoadjuvant versus adjuvant systemic treatment in breast cancer: a meta-analysis. J Natl Cancer Inst.

[4] Fisher B (1997). Effect of preoperative chemotherapy on local-regional disease in women with operable breast cancer: findings from National Surgical Adjuvant Breast and Bowel Project B-18. J Clin Oncol.

[5] Eiermann W (2011). Phase III study of doxorubicin/cyclophosphamide with concomitant versus sequential docetaxel as adjuvant treatment in patients with human epidermal growth factor receptor 2-normal, node-positive breast cancer: BCIRG-005 trial. J Clin Oncol.

[6] Grann VR (2005). Hormone receptor status and survival in a population-based cohort of patients with breast carcinoma. Cancer.

[7] Davies C (2011). Relevance of breast cancer hormone receptors and other factors to the efficacy of adjuvant tamoxifen: patient-level meta-analysis of randomised trials. Lancet.

[8] Peto R (2012). Comparisons between different polychemotherapy regimens for early breast cancer: meta-analyses of long-term outcome among 100,000 women in 123 randomised trials. Lancet.

[9] Paik S (2006). Gene expression and benefit of chemotherapy in women with node-negative, estrogen receptor-positive breast cancer. J Clin Oncol.

[10] Sparano JA (2015). Prospective validation of a 21-gene expression assay in breast cancer. N Engl J Med.

[11] Albain KS (2010). Prognostic and predictive value of the 21-gene recurrence score assay in postmenopausal women with node-positive, oestrogen-receptor-positive breast cancer on chemotherapy: a retrospective analysis of a randomised trial. Lancet Oncol.

[12] Barcenas CH (2014). Risk of hospitalization according to chemotherapy regimen in early-stage breast cancer J. Clin Oncol.

[13] Bernard-Marty C (2003). Second malignancies following adjuvant chemotherapy: 6-year results from a Belgian randomized study comparing cyclophosphamide, methotrexate and 5-fluorouracil (CMF) with an anthracycline-based regimen in adjuvant treatment of node-positive breast cancer patients. Ann Oncol.

[14] Early Breast Cancer Trialists’ Collaborative Group (EBCTCG) (2005). Effects of chemotherapy and hormonal therapy for early breast cancer on recurrence and 15-year survival: an overview of the randomised trials. Lancet.

[15] Tryfonidis K (2015). Management of locally advanced breast cancer-perspectives and future directions. Nat Rev Clin Oncol.

[16] Goncalves R (2012). Use of neoadjuvant data to design adjuvant endocrine therapy trials for breast cancer. Nat Rev Clin Oncol.

[17] Preece PE (1982). Tamoxifen as initial sole treatment of localised breast cancer in elderly women: a pilot study. Br Med J (Clin Res Ed).

[18] Hind D (2006). Surgery versus primary endocrine therapy for operable primary breast cancer in elderly women (70 years plus). Cochrane Database Syst Rev.

[19] Johnston SJ, Cheung KL (2015). The role of primary endocrine therapy in older women with operable breast cancer. Future Oncol.

[20] Dowsett M (2010). Meta-analysis of breast cancer outcomes in adjuvant trials of aromatase inhibitors versus tamoxifen. J Clin Oncol.

[21] Regan MM (2011). Assessment of letrozole and tamoxifen alone and in sequence for postmenopausal women with steroid hormone receptor-positive breast cancer: the BIG 1-98 randomised clinical trial at 8.1 years median follow-up. Lancet Oncol.

[22] Xu HB, Liu YJ, Li L (2011). Aromatase inhibitor versus tamoxifen in postmenopausal woman with advanced breast cancer: a literature-based meta-analysis. Clin Breast Cancer.

[23] Baum M (2002). Anastrozole alone or in combination with tamoxifen versus tamoxifen alone for adjuvant treatment of postmenopausal women with early breast cancer: first results of the ATAC randomised trial. Lancet.

[24] Eiermann W (2001). Preoperative treatment of postmenopausal breast cancer patients with letrozole: A randomized double-blind multicenter study. Ann Oncol.

[25] Smith IE (2005). Neoadjuvant treatment of postmenopausal breast cancer with anastrozole, tamoxifen, or both in combination: the Immediate Preoperative Anastrozole, Tamoxifen, or Combined with Tamoxifen (IMPACT) multicenter double-blind randomized trial. J Clin Oncol.

[26] Cataliotti L (2006). Comparison of anastrozole versus tamoxifen as preoperative therapy in postmenopausal women with hormone receptor-positive breast cancer: the Pre-Operative “Arimidex” Compared to Tamoxifen (PROACT) trial. Cancer.

[27] Masuda N (2012). Neoadjuvant anastrozole versus tamoxifen in patients receiving goserelin for premenopausal breast cancer (STAGE): a double-blind, randomised phase 3 trial. Lancet Oncol.

[28] Ellis MJ (2001). Letrozole is more effective neoadjuvant endocrine therapy than tamoxifen for ErbB-1- and/or ErbB-2-positive, estrogen receptor-positive primary breast cancer: evidence from a phase III randomized trial. J Clin Oncol.

[29] Ellis MJ, Ma C (2007). Letrozole in the neoadjuvant setting: the P024 trial. Breast Cancer Res Treat.

[30] Semiglazov VF (2005). Neoadjuvant endocrine therapy: exemestane vs tamoxifen in postmenopausal ER+ breast cancer patients (T1-4, N1-2, M0). Proc Am Soc Clin Oncol.

[31] Seo JH, Kim YH, Kim JS (2009). Meta-analysis of pre-operative aromatase inhibitor versus tamoxifen in postmenopausal woman with hormone receptor-positive breast cancer. Cancer Chemother Pharmacol.

[32] Gnant M (2011). Adjuvant endocrine therapy plus zoledronic acid in premenopausal women with early-stage breast cancer: 62-month follow-up from the ABCSG-12 randomised trial. Lancet Oncol.

[33] Pagani O (2014). Adjuvant exemestane with ovarian suppression in premenopausal breast cancer. N Engl J Med.

[34] Ellis MJ (2011). Randomized phase II neoadjuvant comparison between letrozole, anastrozole, and exemestane for postmenopausal women with estrogen receptor-rich stage 2 to 3 breast cancer: clinical and biomarker outcomes and predictive value of the baseline PAM50-based intrinsic subtype—ACOSOG Z1031. J Clin Oncol.

[35] Dixon JM (2009). Increase in response rate by prolonged treatment with neoadjuvant letrozole. Breast Cancer Res Treat.

[36] Llombart-Cussac A (2012). Phase II trial with letrozole to maximum response as primary systemic therapy in postmenopausal patients with ER/PgR[+] operable breast cancer. Clin Transl Oncol.

[37] Fontein DB (2014). Efficacy of six month neoadjuvant endocrine therapy in postmenopausal, hormone receptor-positive breast cancer patients—a phase II trial. Eur J Cancer.

[38] Carpenter R (2014). Optimum duration of neoadjuvant letrozole to permit breast conserving surgery. Breast Cancer Res Treat.

[39] Krainick-Strobel UE (2008). Neoadjuvant letrozole in postmenopausal estrogen and/or progesterone receptor positive breast cancer: a phase IIb/III trial to investigate optimal duration of preoperative endocrine therapy. BMC Cancer.

[40] Olson JA (2009). Improved surgical outcomes for breast cancer patients receiving neoadjuvant aromatase inhibitor therapy: results from a multicenter phase II trial. J Am Coll Surg.

[41] Semiglazov VF (2007). Phase 2 randomized trial of primary endocrine therapy versus chemotherapy in postmenopausal patients with estrogen receptor-positive breast cancer. Cancer.

[42] Alba E (2012). Chemotherapy (CT) and hormonotherapy (HT) as neoadjuvant treatment in luminal breast cancer patients: results from the GEICAM/2006-03, a multicenter, randomized, phase-II study. Ann Oncol.

[43] Palmieri C (2014). NEOCENT: a randomised feasibility and translational study comparing neoadjuvant endocrine therapy with chemotherapy in ER-rich postmenopausal primary breast cancer. Breast Cancer Res Treat.

[44] Cortazar P (2014). Pathological complete response and long-term clinical benefit in breast cancer: the CTNeoBC pooled analysis. Lancet.

[45] Gerdes J (1983). Production of a mouse monoclonal antibody reactive with a human nuclear antigen associated with cell proliferation. Int J Cancer.

[46] Senkus E (2013). Primary breast cancer: ESMO Clinical Practice Guidelines for diagnosis, treatment and follow-up. Ann Oncol.

[47] Esposito A, Criscitiello C, Curigliano G (2015). Highlights from the 14(th) St Gallen International Breast Cancer Conference 2015 in Vienna: Dealing with classification, prognostication, and prediction refinement to personalize the treatment of patients with early breast cancer. Ecancermedicalscience.

[48] Ellis MJ (2003). Letrozole inhibits tumor proliferation more effectively than tamoxifen independent of HER1/2 expression status. Cancer Res.

[49] Thurlimann B (2005). A comparison of letrozole and tamoxifen in postmenopausal women with early breast cancer. N Engl J Med.

[50] Goss PE (2013). Exemestane versus anastrozole in postmenopausal women with early breast cancer: NCIC CTG MA.27—a randomized controlled phase III trial. J Clin Oncol.

[51] Dowsett M (2006). Proliferation and apoptosis as markers of benefit in neoadjuvant endocrine therapy of breast cancer. Clin Cancer Res.

[52] Dowsett M (2007). Prognostic value of Ki67 expression after short-term presurgical endocrine therapy for primary breast cancer. J Natl Cancer Inst.

[53] Dowsett M (2011). Endocrine therapy, new biologicals, and new study designs for presurgical studies in breast cancer. J Natl Cancer Inst Monogr.

[54] Ellis MJ (2008). Outcome prediction for estrogen receptor-positive breast cancer based on postneoadjuvant endocrine therapy tumor characteristics. J Natl Cancer Inst.

[55] Hofmann D (2013). WSG ADAPT - adjuvant dynamic marker-adjusted personalized therapy trial optimizing risk assessment and therapy response prediction in early breast cancer: study protocol for a prospective, multi-center, controlled, non-blinded, randomized, investigator initiated phase II/III trial. Trials.

[56] Ueno T (2014). Evaluating the 21-gene assay Recurrence Score(R) as a predictor of clinical response to 24 weeks of neoadjuvant exemestane in estrogen receptor-positive breast cancer. Int J Clin Oncol.

[57] Turnbull AK (2015). Accurate Prediction and Validation of Response to Endocrine Therapy in Breast Cancer. J Clin Oncol.

[58] Ma CX (2015). Mechanisms of aromatase inhibitor resistance. Nat Rev Cancer.

[59] Baselga J (2012). Everolimus in postmenopausal hormone-receptor-positive advanced breast cancer. N Engl J Med.

[60] Piccart M (2014). Everolimus plus exemestane for hormone-receptor-positive, human epidermal growth factor receptor-2-negative advanced breast cancer: overall survival results from BOLERO-2dagger. Ann Oncol.

[61] Baselga J (2009). Phase II randomized study of neoadjuvant everolimus plus letrozole compared with placebo plus letrozole in patients with estrogen receptor-positive breast cancer. J Clin Oncol.

[62] (2012). Comprehensive molecular portraits of human breast tumours. Nature.

[63] Asghar U (2015). The history and future of targeting cyclin-dependent kinases in cancer therapy. Nat Rev Drug Discov.

[64] Finn RS (2015). The cyclin-dependent kinase 4/6 inhibitor palbociclib in combination with letrozole versus letrozole alone as first-line treatment of oestrogen receptor-positive, HER2-negative, advanced breast cancer (PALOMA-1/TRIO-18): a randomised phase 2 study. Lancet Oncol.

[65] Barroso-Sousa R (2013). Biological therapies in breast cancer: common toxicities and management strategies. Breast.

[66] Cui X (2005). Biology of progesterone receptor loss in breast cancer and its implications for endocrine therapy. J Clin Oncol.

[67] Rakha EA (2007). Biologic and clinical characteristics of breast cancer with single hormone receptor positive phenotype. J Clin Oncol.

[68] Mohammed H (2015). Progesterone receptor modulates ERalpha action in breast cancer. Nature.

[69] Kuter I (2012). Dose-dependent change in biomarkers during neoadjuvant endocrine therapy with fulvestrant: results from NEWEST, a randomized Phase II study. Breast Cancer Res Treat.

[70] Di LA (2014). Final overall survival: fulvestrant 500 mg versus 250 mg in the randomized CONFIRM trial. J Natl Cancer Inst.

